# Tumor-derived Exosomal ENO2 Modulates Polarization of Tumor-associated Macrophages through Reprogramming Glycolysis to Promote Progression of Diffuse Large B-cell Lymphoma

**DOI:** 10.7150/ijbs.91154

**Published:** 2024-01-01

**Authors:** Ruonan Shao, Chengcheng Liu, Ruifeng Xue, Xinpei Deng, Lingrui Liu, Cailu Song, Jindong Xie, Hailin Tang, Wenjian Liu

**Affiliations:** 1 State Key Laboratory of Oncology in South China, Guangdong Provincial Clinical Research Center for Cancer, Sun Yat-Sen University Cancer Center, Guangzhou 510060, P. R. China.; 2 Department of Hematology, The Third Affiliated Hospital of Sun Yat‑Sen University, Guangzhou 510630, P. R. China.

**Keywords:** Exosome, ENO2, Macrophage polarization, Glycolysis, Diffuse large B-cell lymphoma, Progression

## Abstract

Macrophages can be polarized into functional classically activated (M1) or alternatively activated (M2) phenotype. Tumor-associated macrophages (TAMs) mainly exhibit M2 phenotype. Previous works determined that up-regulation of enolase 2 (ENO2) in diffuse large B-cell lymphoma (DLBCL) cells can promote macrophages to an M2-like phenotype, thereby consequently promoting the progression of DLBCL. Exosomes are a subset of extracellular vesicles, carrying various bioactive molecules, mediate signals transduction and regulate immune cells. In our study, we investigated the role and related mechanisms of DLBCL-derived exosomal ENO2 in regulating macrophage polarization during DLBCL progression *via* bioinformatics analysis and a series of experiments. The results of bioinformatics analysis indicated that high expression of *ENO2* was positively correlated with DLBCL progression and macrophages M2/M1 ratio. ENO2 protein levels were increased in the exosomes of the sera of DLBCL patients and DLBCL cells. Moreover, the DLBCL-derived exosomes were assimilated by macrophages and then regulated macrophage polarization. The results of *in vitro* and *in vivo* experiments showed that DLBCL-derived exosomal ENO2 modulated macrophages polarization (increased M2 phenotype and decreased M1 phenotype), thereby promoting DLBCL proliferation, migration, and invasion. We then revealed that the modulation of macrophages polarization by DLBCL-derived exosomal ENO2 depended on glycolysis and was promoted through GSK3β/β-catenin/c-Myc signaling pathway. These findings suggested that DLBCL-derived exosomal ENO2 accelerated glycolysis *via* GSK3β/β-catenin/c-Myc signaling pathway to ultimately promote macrophages to an M2-like phenotype, which can promote the proliferation, migration and invasion of DLBCL, suggesting that exosomal ENO2 may be a promising therapeutic target and prognostic biomarker for DLBCL.

## Introduction

Diffuse large B-cell lymphoma (DLBCL) is a disease with significant biological and clinical diversity, constituting approximately 30%-40% of adult non-Hodgkin lymphomas (NHL) 1. CHOP is the preferred chemotherapy regimen for DLBCL. The application of anti-CD20 monoclonal antibody rituximab has significantly improved the overall survival (OS) rate 2-4. Despite these advances, treatment fails in approximately 20%-40% cases, especially in patients with relapse and/or resistance 5. Hence, seeking novel therapeutic targets is of considerable importance.

Macrophages populate the tumor microenvironment (TME) of most tumors and play a key role in tumor immunity. Macrophages display adaptability in response to various stimuli and can be differentiated into unique functional phenotypes, namely, classical activation (M1) or alternative activation (M2) 6-10. M1-like macrophages can kill invading pathogens and tumor cells by secreting proinflammatory cytokines, including interleukin (IL)-12p40 and tumor necrosis factor-α (TNF-α) 9-11. Conversely, M2-like macrophages can promote immunosuppression and immune escape in tumor progress by releasing anti-inflammatory cytokines, including IL-10 and transforming growth factor-β (TGF-β), and exhibiting a high expression of mannose receptor (CD206) 9-11. While tumor-associated macrophages (TAMs) are known to encompass a range of phenotypes, they predominantly display M2-like phenotypes and execute functions that facilitate tumor progression, invasion, metastasis, and immune evasion 9-13, especially in the case of DLBCL 14-16.

Enolase 2 (ENO2) is a 433-amino-acids acidic dimeric protein that is crucial for glycolysis. ENO2 is abnormally expressed in neurogenic and neuroendocrine tumors. Elevated levels of serum ENO2 were found in 17%-21% of NHL 17. Previous studies demonstrated DLBCL exhibits elevated serum ENO2 levels and high serum ENO2 values are closely correlated with unfavorable prognosis of DLBCL 18-19. Moreover, the up-regulation of ENO2 in DLBCL cells can promote macrophages to an M2-like phenotype, thereby subsequently facilitating the progression of DLBCL 20. However, the signals from DLBCL cells for eliciting functional macrophages polarization are not fully understood.

Exosomes (30-200 nm) are a subset of extracellular vesicles carrying a variety of bioactive molecules, including proteins, lipids, RNAs, and DNAs 21. Exosomes mediate signals transduction and regulate immune cells in TME 22-23. However, the regulation of macrophages polarization by DLBC-derived exosomes and its specific mechanisms are still not understood. In the current study, we researched the role and related mechanisms of DLBCL-derived exosomal ENO2 in regulating macrophages polarization during DLBCL progression.

## Materials and methods

### Patients and clinical samples

The micro-arrays and matched clinical data of four cohorts, including GSE10846 (414 patients), GSE32918 (172 patients), GSE119214 (137 patients) and GSE9732 (191 patients) were acquired from the GEO database (http://www.ncbi.nlm.nih.gov/geo/). GSE10846 and GSE32918 include DLBCL patients, GSE119214 includes patients with follicular lymphoma (FL), and GSE9732 includes patients with “normal contributors (NCs), indolent lymphoma (IDL), mantle cell lymphoma (MCL), and DLBCL. Log2 transformation was conducted to normalize the gene expression profiles. The invading scores of immune cells were computed using the ESTIMATE algorithm of “estimate” package. The Human Protein Atlas (https://www.proteinatlas.org) supplied immunohistochemical (IHC) data of ENO2. All DLBCL samples were obtained from Sun Yat-Sen University Cancer Center (SYSUCC). This research was endorsed by the Institutional Review Board and the ethics committees of SYSUCC. Written informed consents were acquired from all contributors prior to treatment to gain blood samples and medical information. The research was conducted following the directions of the Declaration of Helsinki.

### Cell culture and treatment

The human DLBCL cells OCI-LY1, OCI-LY3, SU-DHL-2, SU-DHL-4, human monocyte cell THP-1, and human embryonic kidney cell HEK293T were gained from the American Type Culture Collection and cultured as per manufacturer's protocol. Stimulating by PMA (100 ng/ml, Sigma) for 48 h, THP-1 cells were differentiated toward THP-1 macrophages. The THP-1 macrophages were transformed from suspension to adherent cells and were collected for further research.

THP-1 macrophages were stimulated by different DLBCL-derived exosomes with different ENO2 levels (100 μg/ml protein equivalents) for 24 h. In the mechanisms research, THP-1 macrophages were treated with 2-Deoxy-D-glucose (2-DG, Selleck; 4, 6, and 8 mM), β-catenin activator SKL2001 (20 µM, Selleck) and inhibitor ICG001 (5 µM, Selleck) for 1 h and then stimulated by DLBCL-derived exosomes for 24 h. The supernatant of THP-1 macrophages was collected, purified (0.22 μM, Millex-GP), and then ultracentrifuged to remove cells. The remaining THP-1 macrophages were trypsinized and collected.

Peripheral blood mononuclear cells (PBMCs) were separated from blood samples of contributors by density gradient centrifugation technique containing Ficoll-Paque Premium. The purified B-lymphocytes were extracted from PBMCs using Easy Sep™ direct human B-cell isolation kit (Miltenyi Biotec).

### Isolation and identification of exosomes

Exosomes were extracted from the plasma of DLBCL patients and healthy donors or the supernatant of each DLBCL cell line after 48 h culture. Briefly, collected culture supernatant or plasma samples underwent differential centrifugation at 300, 1200, and 10 000 g for 10, 20, and 30 min in 4 °C, respectively. Afterwards, the supernatant was ultracentrifugally separated under 4 °C and 100 000 g for 3 h. After discarding the supernatant, the pelleted exosomes were rinsed with abundant chilled PBS and subjected to another 3 h of ultracentrifugation at 4 °C and 100 000 g. Finally, the exosome-enriched pellets were redissolved in aseptic PBS.

A NanoSight LM10 (NanoSight) was used for NTA measurements, which is a technique for measuring the particle size and number of exosomes. Exosomes were resuspended and diluted in PBS at a ratio of 1:100 prior to the analysis. Data were captured and analyzed by NTA Version 2.3 (NanoSight). Exosome-enriched pellets were redissolved in PBS buffer and placed on a carbon-coated copper grid, followed by staining with 2% sodium phosphotungstate. A transmission electron microscope (TEM) was employed to observe the samples.

### Exosome labeling and uptake assay

Exosomes were stained with PKH-67 (Sigma), a fluorescent dye, following the protocol and were incubated with unlabeled THP-1 macrophages for 4 h. After harvesting and rinsing twice with PBS, the cells were fixed with 4% paraformaldehyde. With 0.1% Triton-100, cell membranes were ruptured and for 15 min, stained with DAPI (Thermo Fisher Scientific, 10 μg/ml) and phalloidin TRITC (Thermo Fisher Scientific, 10 μg/ml). Images were acquired with an olympus fluorescence confocal microscope.

### Western blot

Total protein extracted from cells or exosome samples were measured by BCA Protein Assay Kit (Thermo Fisher Scientific). Subsequently, the aliquots underwent separation *via* 10% Sodium Dodecyl Sulfate-Polyacrylamide Gel Electrophoresis (SDS-PAGE) and were transferred onto PVDF membranes. These membranes, containing protein extracts, were probed with a specific primary antibody, followed by a secondary antibody. Signal detection was achieved with the aid of Enhanced Chemiluminescence Plus reagents (Millipore). The Nuclear and Cytoplasmic Protein Extraction Kit (Beyotime) was employed to differentiate between cytoplasmic and nuclear proteins, which were subsequently gathered for western blot analysis.

GAPDH was served as the internal benchmark of cellular proteins, CD9 was served as the internal benchmark of exosomal proteins, and Lamin B was served as the internal benchmark of nuclear proteins. The primary antibodies comprised ENO2 (CST), c-Myc (CST), p-GSK3β (Ser9) (CST), Lamin B (CST), β-catenin (Proteintech), GAPDH (Proteintech), and CD9 (Proteintech). The western blot bands were quantitatively by digital image analysis using the Image Pro-Plus software.

### Quantitative real-time polymerase chain reaction (qPCR)

Total RNA, utilized for qPCR assay, was extracted from cells using the TRIzol reagent (Thermo Fisher Scientific) following the manufacturer's protocol. PrimeScript™ RT Reagent Kit (TaKaRa) was employed for the synthesis of complementary DNA (cDNA) from total RNA. Subsequently, qPCR was conducted using the SYBR® Premix Ex Taq™ II Kit (TaKaRa), adhering to the manufacturer's protocol. The primers employed in the qPCR assay are detailed in **Supplementary Table S1**. The calculation of relative gene expression was performed using the 2^-ΔΔCT^ method.

### Lentiviral infection and screening of stable cell lines

The high-titre stocks of lentiviral vectors were generated in HEK293T cells through liposomal transfection, utilizing the modified transfer vector HBLV-CMVIE-ZsGreen-T2A-puro and the packaging vectors pSPAX2 and pMD2G. The viral entities were collected and enriched through the process of ultracentrifugation. DLBCL cells were exposed to the recombinant viral particles in an environment containing 6 µg/ml of polybrene (Sigma). Cells transfected with an empty vector served as the control group. After a 5-day period of expansion and maintenance, the infected cells exhibiting resistance to puromycin (2 µg/ml; Sigma) were selectively chosen. Finally, ENO2-overexpressing (ENO2-OE), ENO2-knockdown (ENO2-sh1, ENO2-sh2) and ENO2-control (ENO2-vector) stably transfected DLBCL cells were established. And the corresponding exosomes from ENO2 stably transfected DLBCL cells were exo/OE, exo/sh1, exo/sh2 and exo/vector, respectively. The shRNA sequences are summaried in **Supplementary Table S2.**

### Cytokine and cell surface marker measurement by ELISA and flow cytometry

THP-1 macrophages were stimulated by different exosomes (100 μg/ml protein equivalents) for 24 h, and the supernatant and THP-1 macrophages were then gathered. The concentrations of IL-12p40 and IL-10 in culture supernatant were determined by enzyme-linked immunosorbent assay (ELISA) kits (Abcam) as per the instructions. And then the THP-1 macrophages were incubated with FITC-conjugated anti-human CD206 (BD Pharmingen) and PE-conjugated anti-human CD68 (BD Pharmingen). The cells were collected and analyzed with a flow cytometry.

### Cell proliferation, migration, and invasion assays

The supernatant of THP-1 macrophages stimulated by exosomes (100 μg/ml protein equivalents) derived from DLBCL cells for 24 h was collected and then used for the cultivation of DLBCL cells.

For cell proliferation assay, DLBCL cells (10^4^ cells/well) were distributed into 96-well plates and cultured with 100 μl of different supernatant mentioned above. The CCK8 (10 μl, Dojindo) was introduced to each well at 0, 24, 48, and 72 h. Following a 2-hour incubation period, the optical density (OD) was measured using a microplate reader at a wavelength of 450 nm, and the proliferation curve was plotted.

For the migration and invasion assays, we utilized transwell chambers equipped with a fluorescence-blocking filter insert. In the migration assay, DLBCL cells (5×10^5^ cells) resuspended in 1 ml of different supernatant mentioned above were positioned in the upper chamber of each insert. For the invasion assay, DLBCL cells (2 × 10^6^ cells) resuspended in 1 ml of different supernatant mentioned above were placed on the upper chamber of each insert coated with matrigel (2 μg/μl, Coring), and 2 ml of RPMI 1640 medium with 20% FBS was introduced to the lower chambers. DLBCL cells migrated at 37 °C for 12 h (migration assay) or 24 h (invasion assay). At the end of migration and invasion, cells that had migrated to the lower chambers were counted under a light microscope using a blood cell counting chamber and normalized to the number of cells present in the regular plate.

### Glucose consumption, ATP and lactate production measurement

THP-1 macrophages were stimulated by different DLBCL-derived exosomes (100 μg/ml protein equivalents, for 24 h), and the supernatant and THP-1 macrophages were gathered for glucose consumption, ATP, and lactate measurement. Glucose and lactate concentrations in culture supernatant were measured with Glucose Test Kit (Jiancheng) and Lactate Assay Kit (Jiancheng). The remaining cells were counted after trypsinization. The levels of glucose consumption and lactate production were normalized to mM/10^5^ cells. For the measurement of intracellular ATP, cell lysates were analyzed using a luciferase-based ATP Assay Kit (Beyotime) as per the instructions, and the ATP production levels were standardized to μM/105 cells.

### Extracellular acidification rate (ECAR) and oxygen consumption rate (OCR)

ECAR and OCR were analysed using an XFe96 analyzer (Seahorse Bioscience) following the manufacturer's protocol. In brief, ECAR involved four sequential phases: basal (drug-free), glycolysis initiation (10 mM glucose), maximal glycolysis initiation (1 μM oligomycin), and glycolysis suppression (50 mM 2-DG). OCR also involved of four sequential phases: basal (drug-free), respiration suppression (1 μM oligomycin), maximal respiration initiation (1 μM fluorocarbonyl cyanide phenylhydrazone), and respiration suppression (0.5 μM Rotenone/AA).

### DLBCL xenograft model

DLBCL-ENO2-sh1 cells (10^7^ cells) were subcutaneously implanted into female nude mice. After one week of implantation, the mice were administered with different exosomes (100 μg protein equivalents in 100 μl PBS) *via* the lateral tail vein every other day for four weeks (total of 14 times). The changes were observed every other day, and the DLBCL xenografts sizes were computed using the formula: volume = (π × length × width × height) / 6. Upon conclusion of the experiment, mice were euthanized and xenografts were excised for weighted and immunohistochemical analyses. The procedures were sanctioned by the Animal Research Committee of SYSUCC.

### Immunohistochemistry and histological evaluation

DLBCL xenografts were preserved, encased in paraffin, and sliced into 5 μm thickness. Following the processes of deparaffinization and rehydration, sections were blocked and subjected to IHC staining procedure for CD206 (CST) using an established protocol. Targeted protein was visualized using diaminobenzidine as substrate.

### Statistical analysis

All data are presented as the mean ± SD deviation. The determination of statistical significance was made using a two-tailed student's t-test for either paired or unpaired data. Differences were deemed statistically significant when the P-value was less than 0.05.

## Results

### High *ENO2* expression levels predicted poor prognosis of DLBCL and were associated with increased macrophages M2/M1 ratio in TME

**Figure 1:**
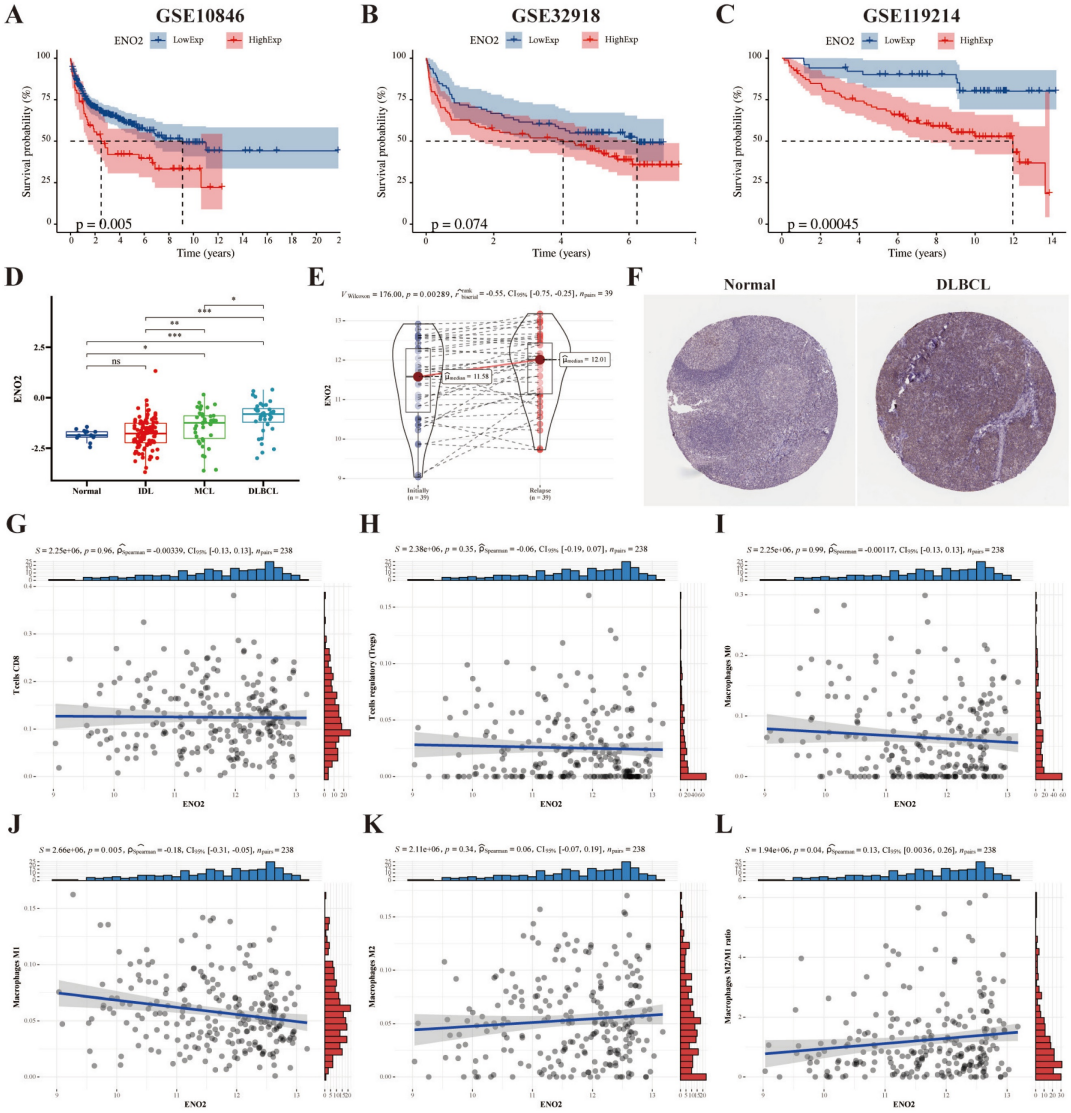
** Bioinformatics analysis identified *ENO2* as a crucial gene for DLBCL prognosis and high *ENO2* expression levels were associated with increased macrophages M2/M1 ratio in TME. A-B**. Kaplan-Meier analysis of OS for DLBCL patients in the GSE10846 and GSE32918 cohorts with high or low *ENO2* expression. **C**. Kaplan-Meier analysis of OS for FL patients in the GSE119214 cohort with high or low *ENO2* expression. **D-E**. Expression signatures of *ENO2* in the GSE9732 and GSE32918 cohorts. **F**. Representative ENO2 IHC staining images from The Human Protein Atlas. **G-L**. Correlation of *ENO2* expression levels with the score of T cells CD8, T cells regulatory, macrophages M0, macrophages M1, macrophages M2 and macrophages M2/M1 ratio. *p<0.05, **p<0.01, ***p<0.001.

We used the DLBCL (GSE10846 and GSE32918), and FL (GSE119214) datasets to explore the prognostic significance of *ENO2*, and the results revealed high *ENO2* expression predicted poor OS of DLBCL and FL (P < 0.05) (**Fig. 1A-C**). *ENO2* expression level was not considerably correlated with stage, ECOG, LDH ratio and age in the GSE10846 cohort (**Supplementary Fig. S1**). The expression levels of *ENO2* were up-regulated in lymphoma compared to normal contributors and escalated in correlation with the increasing invasiveness of the histologic subtype in the GSE9732 dataset (DLBCL > MCL > IDL > NC) (P < 0.05) (**Fig. 1D**). Similarly, relapsed DLBCL had higher *ENO2* expression levels than at initial diagnosis in the GSE32918 dataset (P < 0.05) (**Fig. 1E**). IHC data obtained from The Human Protein Atlas revealed DLBCL tissue had higher *ENO2* levels than normal tissue (**Fig. 1F**). Next, we explored the effects of *ENO2* on immune cells in GSE32918 dataset. Our results suggested that the high *ENO2* expression levels were associated with increased macrophages M2/M1 ratio in TME (P < 0.05) (**Fig. 1G-L**).

### Exosomal ENO2 was elevated in the sera of DLBCL patients and DLBCL cells

**Figure 2:**
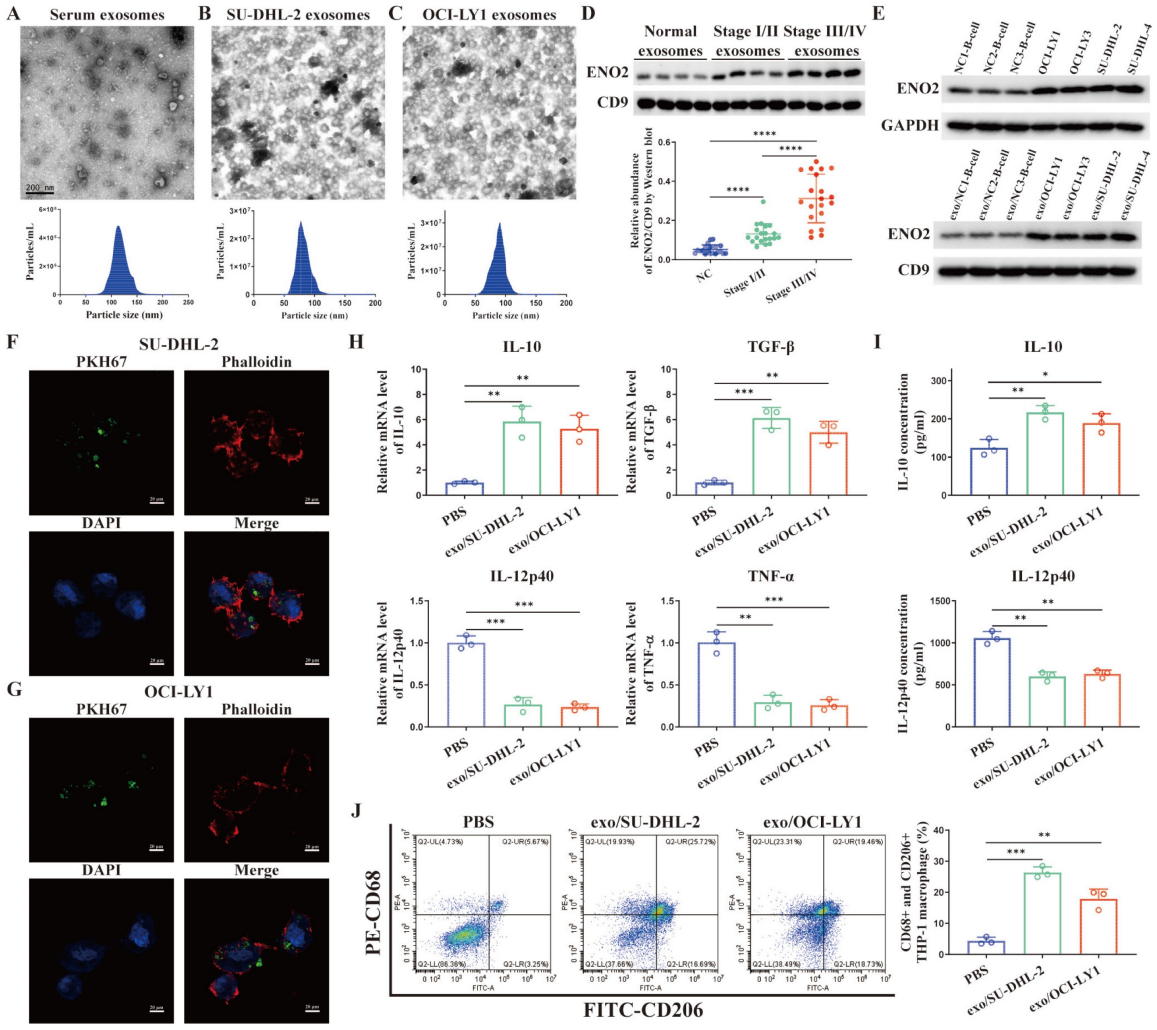
** Exosomal ENO2 was elevated in the DLBCL and DLBCL-derived exosomes regulated macrophages polarization. A-C**. TEM images and NTA showed the structures and sizes of exosomes. **D**. Serum exosomal ENO2 was elevated in the sera of DLBCL patients and related to the progression of DLBCL. **E**. ENO2 protein levels were higher in both cellular and exosomes of DLBCL cells than that of B-lymphocytes from NCs. **F-G**. Confocal microscope images showed that THP-1 macrophages uptake exosomes from DLBCL cells. Exosomes (green), F-actin (red), and Nuclei (blue). **H**. mRNA levels of IL-10, TGF-β, IL-12p40, and TNF-α in THP-1 macrophages. **I**. IL-10 and IL-12p40 levels in supernatant of THP-1 macrophages. **J**. Proportion of CD68+CD206+THP-1 macrophages and quantification analysis. Data were shown as the mean±SD. *p<0.05, **p<0.01, ***p<0.001.

TEM images of exosomes and the sizes of exosomes derived from the serum of DLBCL patients and two DLBCL cells were shown in **Fig. 2A-C**. Exosomes were isolated from the serum of normal contributors (NCs) (n = 20), stage Ⅰ/Ⅱ DLBCL patients (n = 20) and stage III/Ⅳ DLBCL patients (n = 20), respectively. We determined exosomes ENO2 levels by western blot. ENO2 protein levels were higher in serum exosomes of DLBCL patients than that of NCs, and the serum exosomal ENO2 levels were higher in stage ӀӀӀ/ӀV DLBCL patients than that in stage І/ӀӀ DLBCL patients (**Fig. 2D**). In addition, ENO2 protein levels were higher in both exosomes and cellular of DLBCL cells than that from B-lymphocytes of NCs (**Fig. 2E**).

### DLBCL-derived exosomes were absorbed by THP-1 macrophages and regulated macrophages polarization *via* ENO2

THP-1 macrophages were exposed to fluorescence-tagged exosomes from DLBCL cells for 4 h to investigate the interactions between DLBCL cells and macrophages through exosomes and verify the absorption of DLBCL-derived exosomes by THP-1 macrophages. As depicted in **Fig. 2F-G**, the labeled exosomes indicated as green dot-like structures were contained within the THP-1 macrophages. THP-1 macrophages were subsequently exposed to exosomes derived from DLBCL cells for 24 h, and the levels of M2 macrophages markers (mRNA levels of IL-10 and TGF-β by qPCR, IL-10 concentration in supernatant by ELISA, CD68 and CD206 expression by flow cytometry) and M1 macrophages markers (mRNA levels of IL-12p40 and TNF-α by qPCR, IL-12p40 concentration in supernatant by ELISA) were detected. In comparison to PBS stimulation, stimulated by exosomes derived from two DLBCL cells notably elevated the levels of M2 macrophages markers and decreased the levels of M1 macrophages markers, as shown in **Fig. 2H-J**.

It is well known that increased M2-like macrophages in TME can promote tumor progression 9-11. Previous studies demonstrated ENO2, as an unfavorable factor, can promote macrophages to an M2-like phenotype, thereby subsequently facilitating the progression of DLBCL 18-20. Combined all the findings, we speculated DLBCL-derived exosomes can regulate macrophages polarization *via* ENO2. To verify this hypothesis, we established ENO2 stably transfected DLBCL cells for further verification.

**Figure 3:**
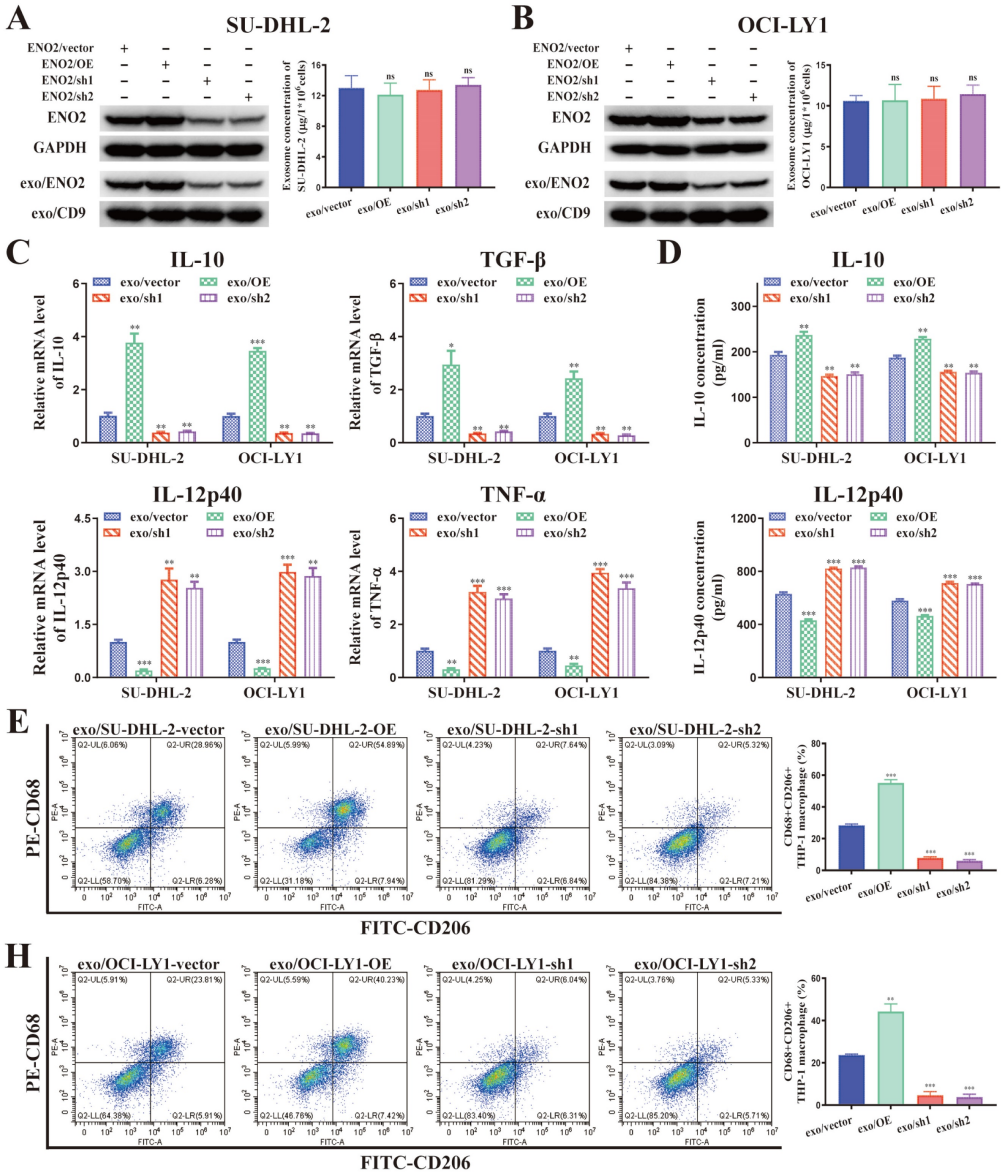
** DLBCL-derived exosomes regulated macrophages polarization *via* ENO2.** THP-1 macrophages were treated with exosomes from different ENO2 stably transfected DLBCL cells and then macrophages polarization markers were detected. **A-B**. ENO2 stably transfected DLBCL cells were established. No significant difference was observed in the concentration of exosomes from different stably transfected DLBCL cells. **C**. mRNA levels of IL-10, TGF-β, IL-12p40, and TNF-α in THP-1 macrophages. **D**. IL-10 and IL-12p40 levels in supernatant of THP-1 macrophages. **E-H**. Proportion of CD68+CD206+THP-1 macrophages and quantification analysis. Data were shown as the mean±SD. *p<0.05, **p<0.01, ***p<0.001.

A positive correlation was noted between cellular and exosomal ENO2 expression in ENO2 stably transfected DLBCL cells (**Fig. 3A-B**). There was no notable difference in the concentration of exosomes released from various ENO2 stably transfected DLBCL cells (**Fig. 3A-B**). THP-1 macrophages were then incubated with exosomes from different ENO2 stably transfected DLBCL cells. As we expected, stimulated by exosomes with up-regulated ENO2 significantly elevated the levels of M2 macrophages markers and decreased the levels of M1 macrophages markers, conversely, the down-regulation of ENO2 exhibited the opposite effects (**Fig. 3C-H**).

### DLBCL-derived exosomal ENO2 promoted macrophages to an M2-like phenotype *in vitro* and *in vivo*, which in turn promoted proliferation, migration, and invasion of DLBCL cells

**Figure 4:**
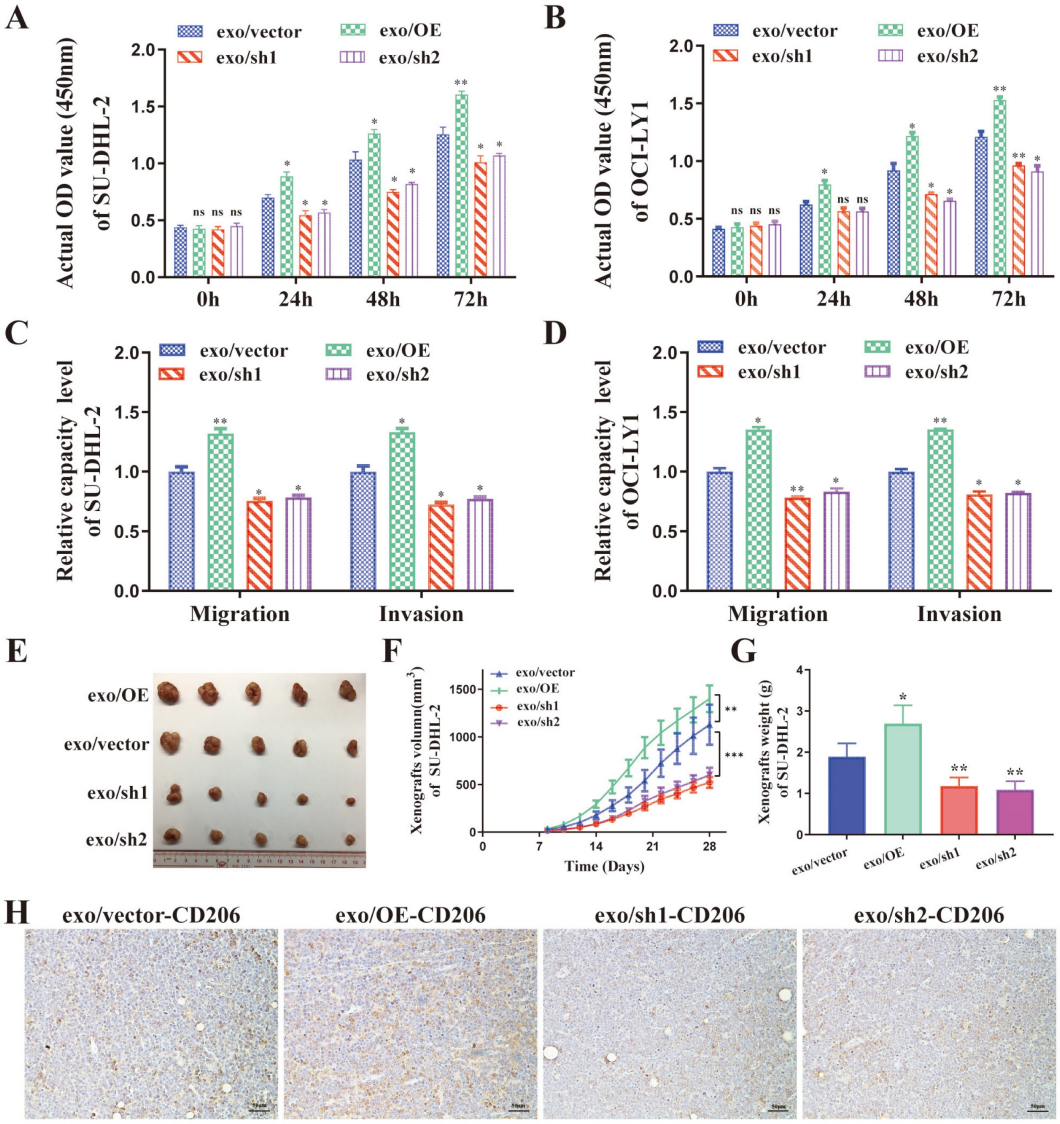
** DLBCL-derived exosomal ENO2 promoted macrophages to an M2-like phenotype *in vivo* and* in vitro*, which in turn promoted proliferation, migration, and invasion of DLBCL cells.** THP-1 macrophages were treated with exosomes from different ENO2 stably transfected DLBCL cells and then the supernatant of THP-1 macrophages treated with different exosomes were collected and used to culture DLBCL cells. **A-B**. CCK8 showing proliferation capacities of DLBCL cells. **C-D**. Transwell assays showing the migration and invasion capacities of DLBCL cells. SU-DHL-2-ENO2-sh1 cells were subcutaneously injected into nude mice, and the mice were then injected with exosomes from different ENO2 stably transfected SU-DHL-2 cells *via* the lateral tail vein every other day for four weeks (total of 14 times). **E**. Tumor volume of SU-DHL-2-ENO2-sh1 xenograft. **F**. Growth speed of SU-DHL-2-ENO2-sh1 xenograft. **G**. Tumor weight of SU-DHL-2-ENO2-sh1 xenograft. **H**. IHC staining images (CD206) of SU-DHL-2-ENO2-sh1 xenograft. Data were shown as the mean±SD. *p<0.05, **p<0.01, ***p <00.001.

The supernatant of THP-1 macrophages stimulated by exosomes from different ENO2 stably transfected DLBCL cells was collected and used to culture DLBCL cells, and the proliferation by CCK8 assays, migration and invasion by transwell chambers in DLBCL cells were then detected. As shown in **Fig. 4A-D**, the supernatant from THP-1 macrophages, stimulated by exosomes with up-regulated ENO2, enhanced the proliferation, migration, and invasion capabilities of DLBCL cells. Conversely, the down-regulation of ENO2 showed the opposite results.

DLBCL-ENO2-sh1 were selected to establish xenograft *in vivo* experiments to further assess the functions of DLBCL-derived exosomal in regulating macrophages polarization and the effects of M2-like macrophages on DLBCL. DLBCL cells (10^7^ cells) were subcutaneously implanted into nude mice, and the mice were subsequently injected with exosomes from different ENO2 stably transfected DLBCL cells (100 μg protein equivalents in 100 μl PBS) *via* the lateral tail vein every other day for four weeks (total of 14 times).

These nude mice injected exosomes with up-regulated ENO2 exhibited a rapid xenograft formation and a significant increase in xenograft sizes and weight, by contrast, injected exosomes with down-regulated ENO2 resulted in delayed xenograft formation and a significant reduction of xenograft sizes and weight (**Fig. 4E-G** and** Supplementary Fig. S2**). The expressions of CD206 in xenografts were also detected by IHC. Consistent with the *in vitro* findings, the proportion of CD206+ cells in the xenografts were increased in mice injected with exosomes with up-regulated ENO2, whereas the proportion of CD206+ cells in the xenografts reduced in mice injected with exosomes with down-regulated ENO2 (**Fig. 4H** and **Supplementary Fig. S2**).

### Regulation of macrophages polarization by DLBCL-derived exosomal ENO2 depended on glycolysis

**Figure 5:**
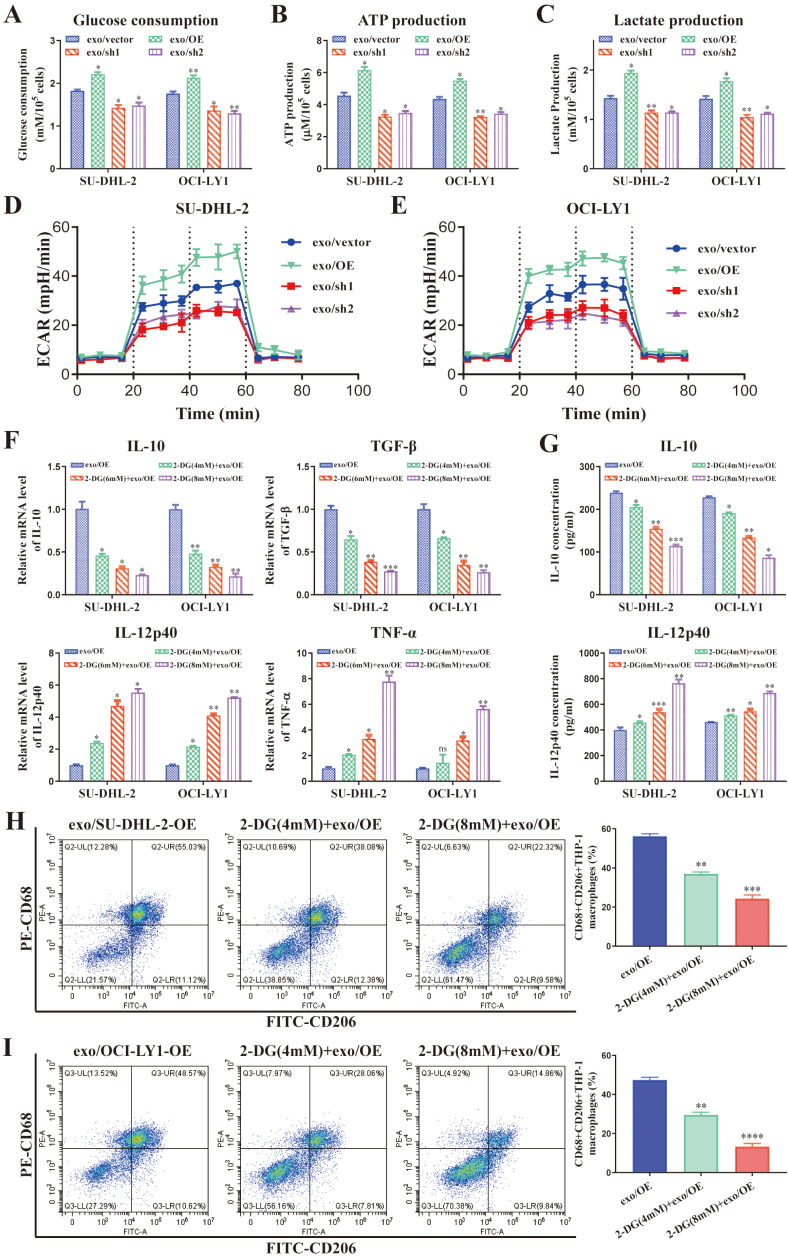
** DLBCL-derived exosomal ENO2 regulated macrophages polarization depended on glycolysis.** THP-1 macrophages were treated with exosomes from different ENO2 stably transfected DLBCL cells and then glycolysis-related indicators were detected. **A**. Glucose consumption of THP-1 macrophages. **B**. ATP production of THP-1 macrophages. **C**. Lactate production of THP-1 macrophages. **D-E**. ECAR of the THP-1 macrophages. THP-1 macrophages were treated with different concentrations of 2-DG for 1 h and then stimulated by exosomes with up-regulated ENO2 for 24 h. Macrophages polarization markers were detected. **F**. mRNA levels of IL-10, TGF-β, IL-12p40, and TNF-α in THP-1 macrophages. **G**. IL-10 and IL-12p40 levels in supernatant of THP-1 macrophages. **H-I**. Proportion of CD68+CD206+THP-1 macrophages and quantification analysis. Data were shown as the mean±SD. *p<0.05, **p<0.01, ***p<0.001.

ENO2 is an essential glycolytic enzyme, and several studies have convincingly demonstrated that the polarization of macrophages can be modulated by glycolysis 11, 24-28. Glucose consumption, glycolysis productions (ATP, lactate), ECAR and OCR were detected in THP-1 macrophages stimulated by exosomes from different ENO2 stably transfected DLBCL cells. Stimulated by exosomes with up-regulated ENO2 significantly promoted glycolysis (increased glucose consumption leads to elevated ATP, lactate production and ECAR) in THP-1 macrophages. By contrast, stimulated by exosomes with down-regulated ENO2 significantly suppressed glycolysis (**Fig. 5A-E** and** Supplementary Fig. S3**).

We applied 2-DG, a type of glucose analogue, as a competitive inhibitor of glycolysis to further confirm regulation of macrophages polarization by DLBCL-derived exosomal ENO2 depended on glycolysis. THP-1 macrophages were treated with 2-DG (4, 6, and 8 mM) for 1 h and then stimulated by exosomes with up-regulated ENO2 for 24 h. The findings indicated that the levels of M2 macrophages markers diminished while that of M1 macrophages markers elevated gradually as the concentrations of 2-DG increased (**Fig. 5F-I**), thereby indicating that 2-DG reversed regulation of macrophages polarization in a dose-dependent fashion. These findings imply that regulation of macrophages polarization by DLBCL-derived exosomal ENO2 depended on glycolysis.

### DLBCL-derived exosomal ENO2 accelerated glycolysis *via* GSK3β/β-catenin/c-Myc signaling pathway, thereby regulating macrophages polarization

Activation of β-catenin/c-Myc signaling can promote macrophages M2 polarization through accelerating glycolysis 11, 28-31. GSK3β phosphorylation at Ser9 can decrease the activity of GSK3β, thereby promoting the nuclear translocation of β-catenin 32. ENO2 enhanced GSK-3β phosphorylation to accelerate glycolysis 33. Moreover, the protein levels of β-catenin and c-Myc were notably elevated in THP-1 macrophages stimulated by exosomes from DLBCL cells (**Supplementary Fig. S4**). Thus, we speculated that DLBCL-derived exosomal ENO2 accelerated glycolysis *via* GSK3β/β-catenin/c-Myc signaling pathway, thereby regulating macrophages polarization.

The protein levels of p-GSK3β (Ser9), β-catenin and c-Myc was detected in THP-1 macrophages stimulated by exosomes from different ENO2 stably transfected DLBCL cells to ascertain the impact of DLBCL-derived exosomal ENO2 on GSK3β/β-catenin/c-Myc signaling pathway.

**Figure 6:**
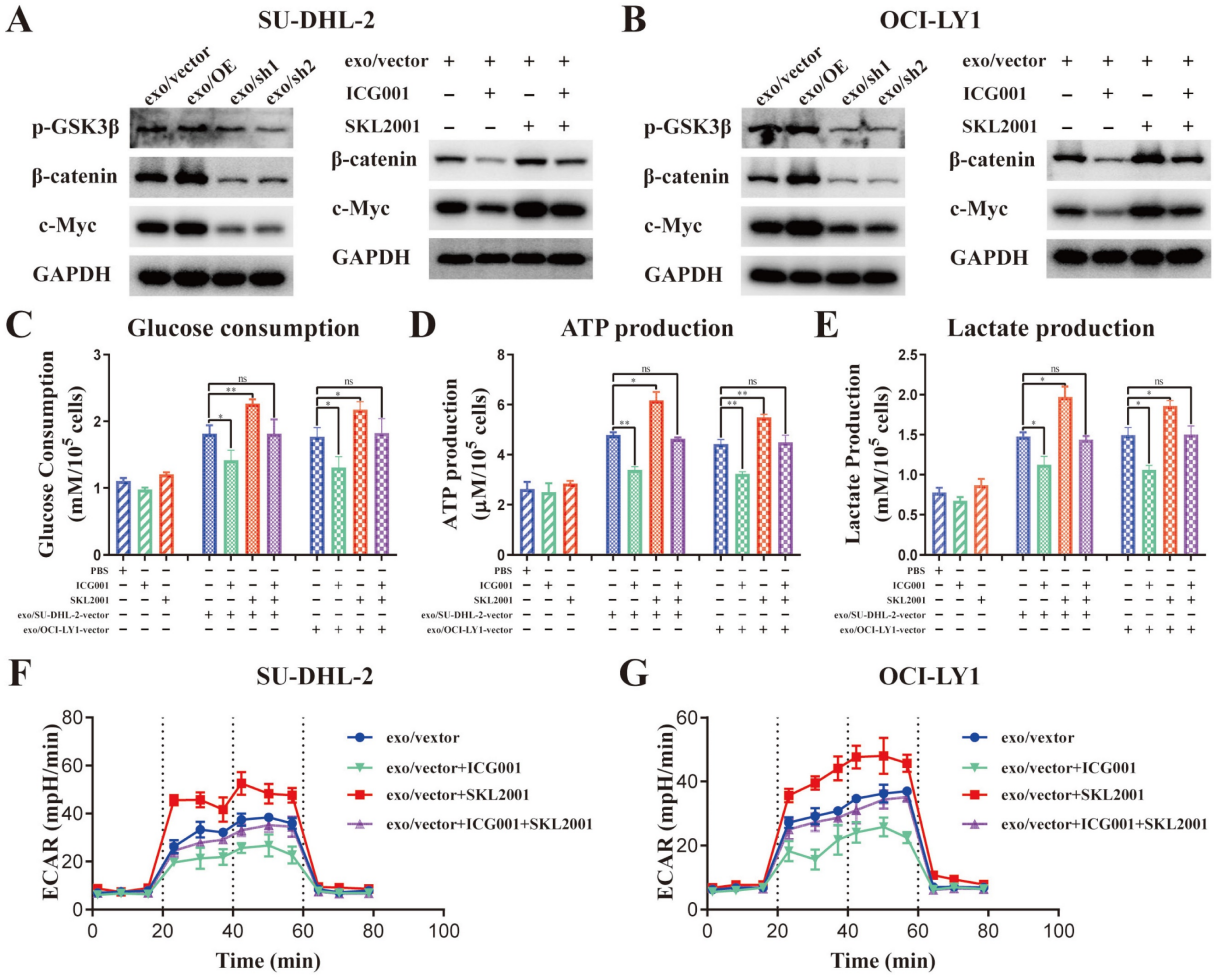
** DLBCL-derived exosomal ENO2 regulated macrophages glycolysis *via* GSK3β/β-catenin/c-Myc signaling pathway. A-B**. The protein expression levels of p-GSK3β (Ser9), β-catenin and c-Myc were detected in THP-1 macrophages stimulated by exosomes from different ENO2 stably transfected DLBCL cells; ICG001 can decrease the protein expression levels of β-catenin and c-Myc in THP-1 macrophages; SKL2001 can increase the protein expression levels of β-catenin and c-Myc in THP-1 macrophages. THP-1 macrophages were treated with ICG001 or SKL2001 for 1 h and then stimulated by exosomes from DLBCL cells for 24 h. Glycolysis-related indicators were detected. **C**. Glucose consumption of THP-1 macrophages. **D**. ATP production of THP-1 macrophages. **E**. Lactate production of THP-1 macrophages. **F-G**. ECAR of THP-1 macrophages. Data were shown as the mean±SD. *p<0.05, **p<0.01, ***p<0.001.

Stimulated by exosomes with up-regulated ENO2 increased the expression of p-GSK3β (Ser9), β-catenin and c-Myc; meanwhile, stimulated by exosomes with down-regulated ENO2 diminished the expression of p-GSK3β (Ser9), β-catenin and c-Myc (**Fig. 6A-B** and** Supplementary Fig. S5**). β-catenin inhibitor ICG001 (5 µM) and activator SKL2001 (20 µM) were used to verify whether the regulation of macrophages polarization by DLBCL-derived exosomal ENO2 was mediated through GSK3β/β-catenin/c-Myc signals. ICG001 can decrease the expression of β-catenin and c-Myc, while SKL2001 can increase the expression of β-catenin and c-Myc (**Fig. 6A-B** and** Supplementary Fig. S5**). In addition, ICG001 can suppress macrophages glycolysis stimulated by DLBCL-derived exosomal ENO2, while SKL2001 can enhance macrophages glycolysis stimulated by DLBCL-derived exosomal ENO2 (**Fig. 6C-G**). These results revealed that DLBCL-derived exosomal ENO2 accelerated glycolysis *via* GSK3β/β-catenin/c-Myc signaling pathway.

**Figure 7:**
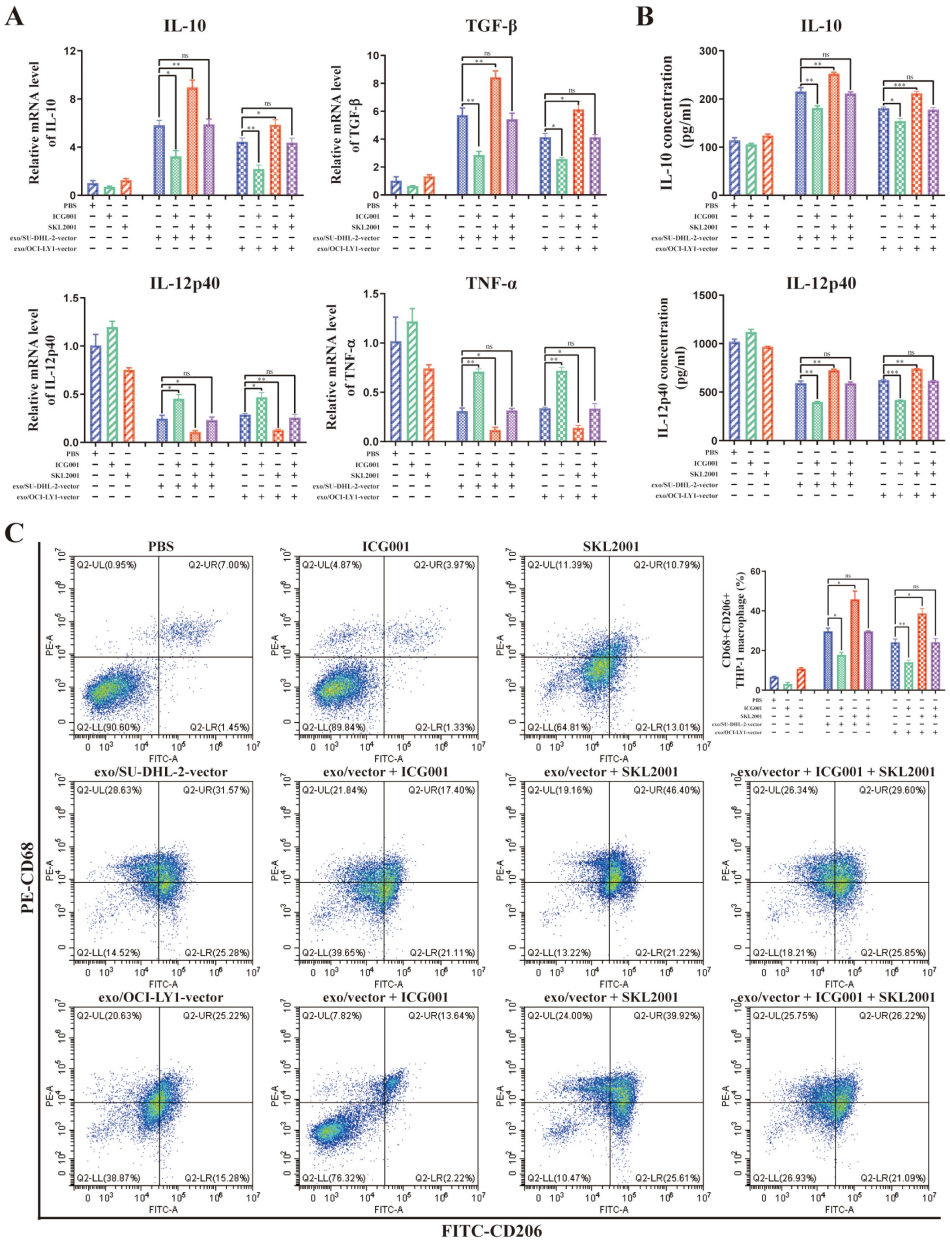
** DLBCL-derived exosomal ENO2 regulated macrophages polarization *via* GSK3β/β-catenin/c-Myc signaling pathway.** THP-1 macrophages were treated with ICG001 or SKL2001 for 1 h and then stimulated by exosomes from DLBCL cells for 24 h. Macrophages polarization markers were detected. **A**. mRNA levels of IL-10, TGF-β, IL-12p40, and TNF-α in THP-1 macrophages. **B**. IL-10 and IL-12p40 levels in supernatant of THP-1 macrophages. **C**. Proportion of CD68+CD206+THP-1 macrophages and quantification analysis. Data were shown as the mean±SD. *p<0.05, **p<0.01, ***p<0.001.

What's more, ICG001 can reverse the modulation of macrophages M2 polarization by DLBCL-derived exosomal ENO2, while SKL2001 can amplify the modulation of macrophages M2 polarization by DLBCL-derived exosomal ENO2 (**Fig. 7A-C**). These findings revealed that DLBCL-derived exosomal ENO2 regulated macrophages polarization *via* GSK3β/β-catenin/c-Myc signaling pathway.

**Figure 8:**
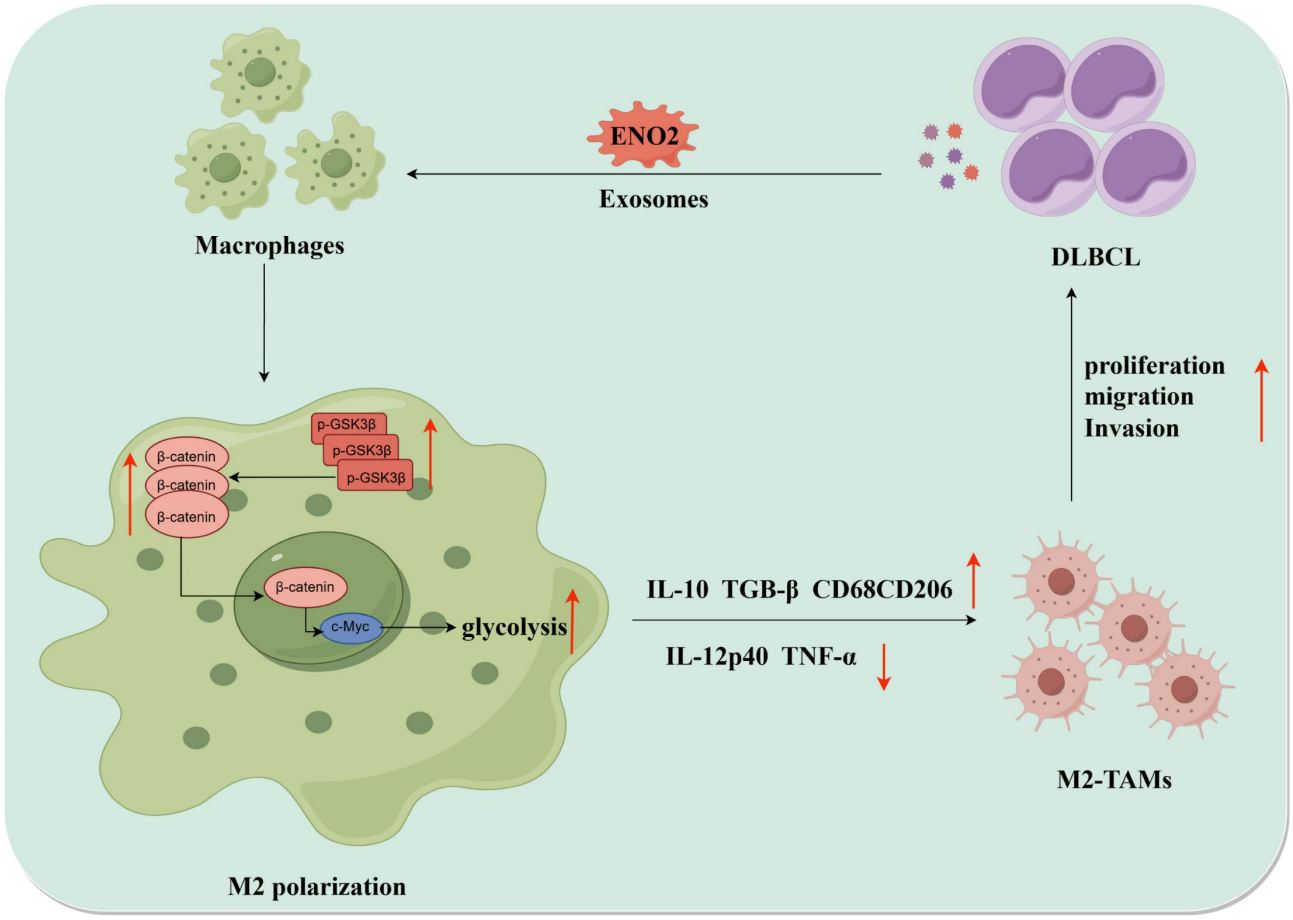
** The diagrammatic representation depicted the positive feedback loop between DLBCL cells and TAMs.** DLBCL-derived exosomes can be absorbed by THP-1 macrophages. DLBCL-derived exosomal ENO2 can up-regulate the phosphorylation of GSK3β (Ser9) of macrophages, and then promoted the expression and nuclear translocation of β-catenin, which can accelerate glycolysis (Glucose consumption, ATP production, Lactate production, ECAR ) by activating c-Myc. These processes promoted macrophages to an M2-like phenotype (increased M2 macrophages markers and decreased M1 macrophages markers), which in turn promoted the proliferation, migration and invasion of DLBCL cells. DLBCL, Diffuse large B-cell lymphoma; TME, Tumor microenvironment; TAMs, Tumour-associated macrophages; ECAR, Extracellular acidification rate.

Collectively, DLBCL-derived exosomal ENO2 accelerated glycolysis *via* GSK3β/β-catenin/c-Myc signal, thereby promoting macrophages to an M2-like phenotype, which in turn facilitated the proliferation, migration and invasion of DLBCL cells. The positive feedback loop between DLBCL cells and TAMs was shown in **Fig. 8**.

## Discussion

TAMs constitute up to 50% of the immune system component in tumor tissues, and M2 phenotype is dominant, particularly in the anoxia area 34. TAMs have been extensively depicted as participating in tumorigenesis and aggressive progression in DLBCL 14-16, 35. The dynamic interaction between the tumor and its TME orchestrates crucial events conducing to tumor progression and drug resistance 36. Signals from the microenvironment are delivered not only through cell-to-cell cooperation but also via various molecules, such as soluble factors (cytokines, chemokines) or exosomes, which play a crucial role in tumor-host crosstalk 37-38. Tumor-derived exosomes serve as an effective platform for transferring soluble crosstalk factors 39-40. The functions of exosomes are contingent on their contents, and consequently, relying on the cell types from which they originate 39-40. The exchange of cellular contents between tumor and TME cells, facilitated by exosomes, plays a pivotal role in modulating the TME, involving lymphomas 41-42. However, information on the modulation of TAMs by lymphoma-derived exosomes is sparse.

ENO2 is overexpressed and has been proposed to predict prognosis in a few malignant tumors 43-44. Previous studies demonstrated that serum ENO2 value was closely correlated with poor prognosis in DLBCL 18-19 and illustrated that ENO2 played a pivotal role in reversing M1 to M2 polarization in macrophages 20. However, the signals to elicit M2 polarization between DLBCL cells and macrophages are not fully understood. Bioinformatics analysis revealed high *ENO2* expression levels predicted poor prognosis of DLBCL and were associated with increased macrophages M2/M1 ratio in TME. Our proteomic approach demonstrated that exosomal ENO2 level was increased in DLBLC cells and DLBLC patients' sera, and the serum exosomal ENO2 level was increased in stage ӀӀӀ/ӀV DLBCL patients than that in stage І/ӀӀ DLBCL patients, thereby indicating that ENO2 can be considered as a marker of DLBCL occurrence and progression. Additionally, a positive correlation was noted between cellular and exosomal ENO2 protein levels.

Several studies have demonstrated the existence and biological function of proteins in exosomes 45-46. This study showed that DLBCL-derived exosomes can be taken up by macrophages and regulate macrophages polarization (increased M2 phenotype and decreased M1 phenotype). Experiments using exosomes from different ENO2 stably transfected DLBCL cells were performed to validate whether DLBCL-derived exosomal ENO2 regulated macrophages polarization. The results revealed DLBCL-derived exosomal ENO2 regulated macrophages polarization, which in ture promoted proliferation, migration, and invasion of DLBCL cells *in vitro* and *in vivo*.

ENO2 is an essential glycolytic enzyme, and glycolysis is known to contribute to macrophages polarization regulation 24-27. We found that DLBCL-derived exosomal ENO2 promoted glycolysis in THP-1 macrophages and 2-DG reversed the regulation of macrophages polarization in a dose-dependent fashion. Therefore, the regulation of macrophages polarization by DLBCL-derived exosomal ENO2 depended on glycolysis. Active molecules derived from tumor cells could activate β-catenin/c-Myc-mediated glycolysis to promote M2 polarization of TAMs 11. Several studies have also shown that activation of the β-catenin/c-Myc signals promoted macrophages M2 polarization 30, 47. GSK3β phosphorylation at Ser9 can promote the nuclear translocation of β-catenin 32. ENO2 enhanced GSK-3β phosphorylation to accelerate glycolysis 33. We proposed that GSK3β/β-catenin/c-Myc signals participate in DLBCL-derived exosomal ENO2-regulated macrophages polarization. As expected, DLBCL-derived exosomal ENO2 could up-regulate the expression of p-GSK3β (Ser9), β-catenin and c-Myc. Treatment of β-catenin inhibitor ICG001 can suppress macrophages glycolysis and M2 polarization stimulated by DLBCL-derived exosomal ENO2. Treatment of β-catenin activator SKL2001 can enhance macrophages glycolysis and M2 polarization stimulated by DLBCL-derived exosomal ENO2. These results revealed that DLBCL-derived exosomal ENO2 accelerated glycolysis *via* GSK3β/β-catenin/c-Myc signaling pathway, thereby promoting macrophages M2 polarization.

In summary, DLBCL-derived exosomes can be taken up by macrophages, and DLBCL-derived exosomal ENO2 accelerated glycolysis *via* GSK3β/β-catenin/c-Myc signaling pathway to regulate macrophages toward an immunosuppression phenotype, which could consequently promote DLBCL progression. The recognition of the pivotal role of DLBCL-derived exosomal ENO2 in modulating TAMs polarization offers underlying molecular targets for DLBCL therapy.

## Supplementary Material

Supplementary figures and tables.

## Abbreviations

DLBCL: diffuse large B-cell lymphoma
NHL: non-Hodgkin lymphomas
TME: tumor microenvironment
IL: interleukin
TNF-α: tumor necrosis factor-α
TGF-β: transforming growth factor-β
TAM: tumor-associated macrophage
ENO2: enolase 2
FL: follicular lymphoma
IDL: indolent lymphoma
MCL: mantle cell lymphoma
IHC: immunohistochemical
2-DG: 2-Deoxy-D-glucose
PBMCs: peripheral blood mononuclear cells
NTA: nanoparticle tracking analysis
TEM: transmission electron microscope
qPCR: quantitative real-time polymerase chain reaction
OE: overexpressing
shRNA: small hairpin RNA
ELISA: enzyme-linked immunosorbent assay
CCK8: Cell Counting Kit-8
OD: optical density
ECAR: extracellular acidification rate
SD: standard deviation
NC: normal contributor
