# Supplementary material for: Tumor-derived Exosomal ENO2 Modulates Polarization of Tumor-associated Macrophages through Reprogramming Glycolysis to Promote Progression of Diffuse Large B-cell Lymphoma
Source: Int J Biol Sci. 2024 Jan 1;20(3):848–63. doi: 10.7150/ijbs.91154 (PMC10797692; doi:10.7150/ijbs.91154)

Table S1. qRT-PCR primers for *IL-10, TGF-β, IL-12p40, TNF-α* and *GAPDH*.

IL-10	Forward Primer	GACTTTAAGGGTTACCTGGGTTG
	Reverse Primer	TCACATGCGCCT-TGATGTCTG
TGF-β	Forward Primer	TACCTGAACCCGTGTTGCTCTC
	Reverse Primer	G-TTGCTGAGGTATCGCCAGGAA
IL-12p40	Forward Primer	CTCTGGCAAAACCCTGACC
	Reverse Primer	GCTTAGAACCTCGCCTCCTT
TNF-α	Forward Primer	AGGCGGTGCTTGTTCCTCAG
	Reverse Primer	GTTATCTCTCAGCTCCACGC
GAPDH	Forward Primer	AGGTCGGTGTGAACGGATTTG
	Reverse Primer	TGTAGACCATGTAGTTGAGGTCA

Table S2. Sequence of *ENO2* shRNAs.

ENO2	shRNA1	CCCACAGTGGAGGTGGATCTCTATA
	shRNA2	CAAGGGAGTCATCAAGGACAA

Fig. S1. Correlation of *ENO2* expression levels with stage, ECOG, LDH ratio and age in the GSE10846 cohort.

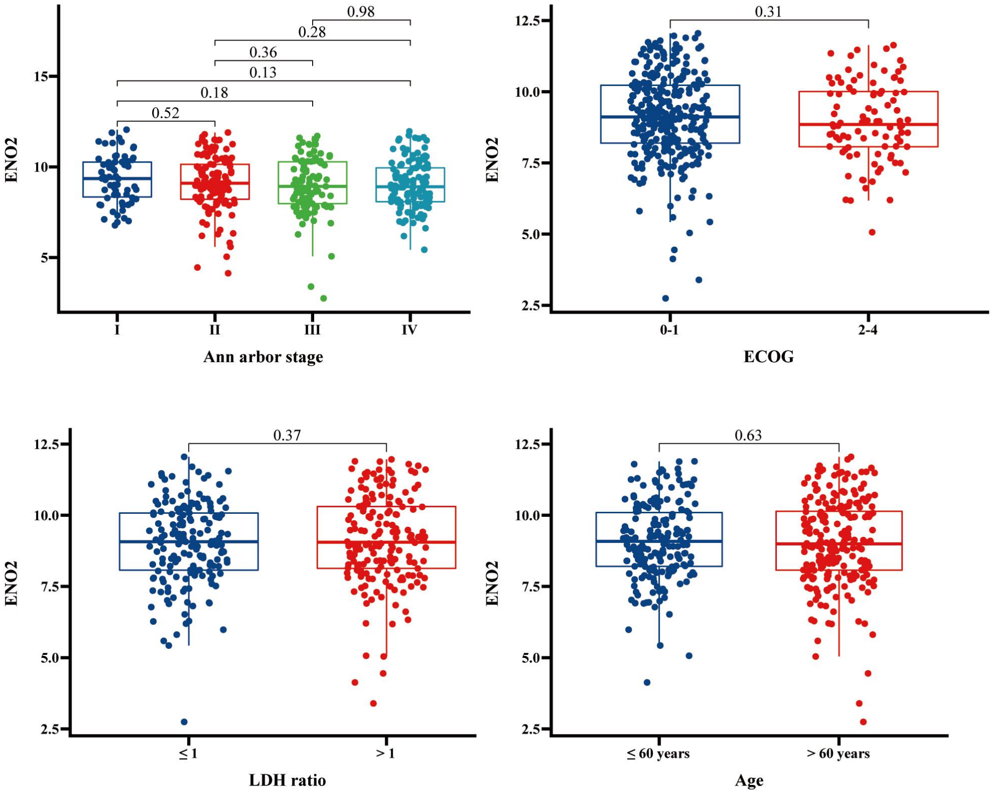

Fig. S2. **A**. Tumor volume of OCI-LY1-ENO2-sh1 xenograft. **B**. Growth speed of OCI-LY1-ENO2-sh1 xenograft. **C**. Tumor weight of OCI-LY1-ENO2-sh1 xenograft. **D**. IHC staining images (CD206) of OCI-LY1-ENO2-sh1 xenograft. Data were shown as the mean±SD. *p＜0.05, **p＜0.01, ***p＜0.001.

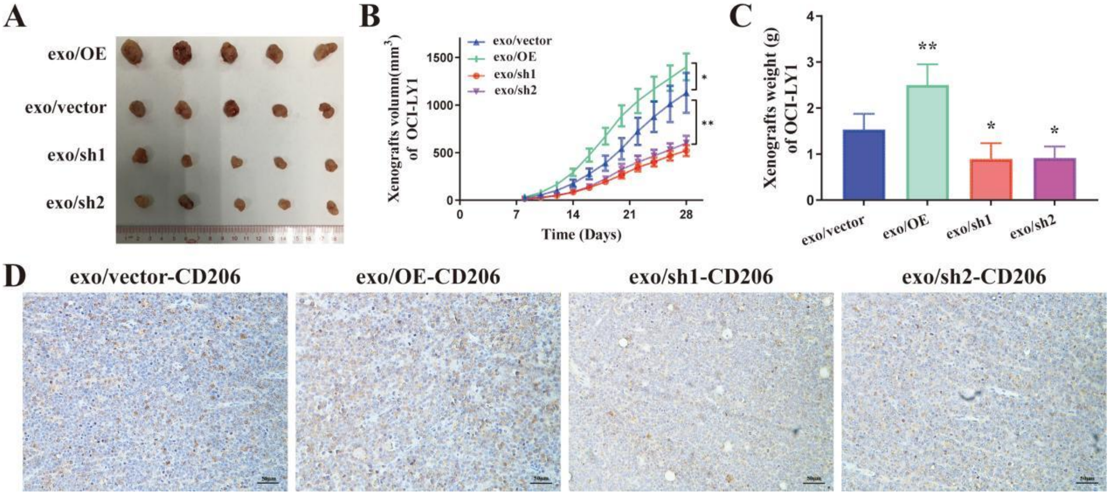

Fig. S3. OCR of the THP-1 macrophages stimulated by exosomes from different ENO2 stably transfected DLBCL cells.

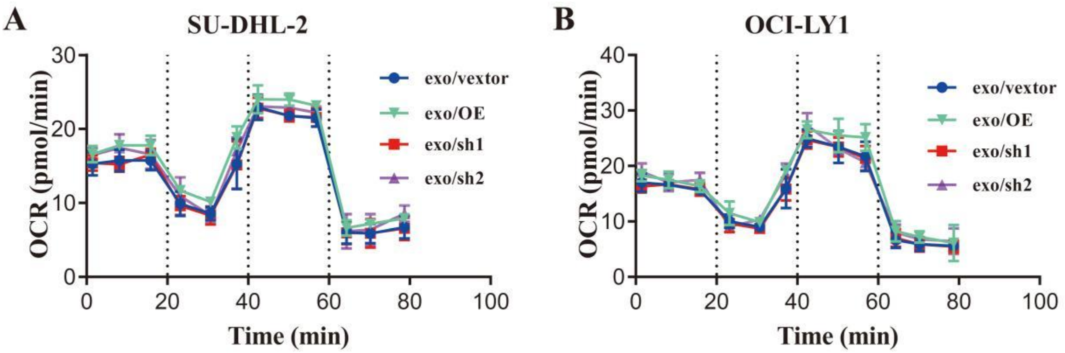

Fig. S4. The protein expression levels of β-catenin and c-Myc were detected in THP-1 macrophages stimulated by PBS or exosomes from DLBCL cells.

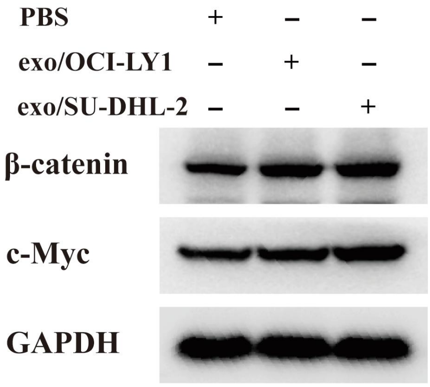

Fig. S5. The protein expression levels of nuclear translocation of β-catenin were detected in THP-1 macrophages.

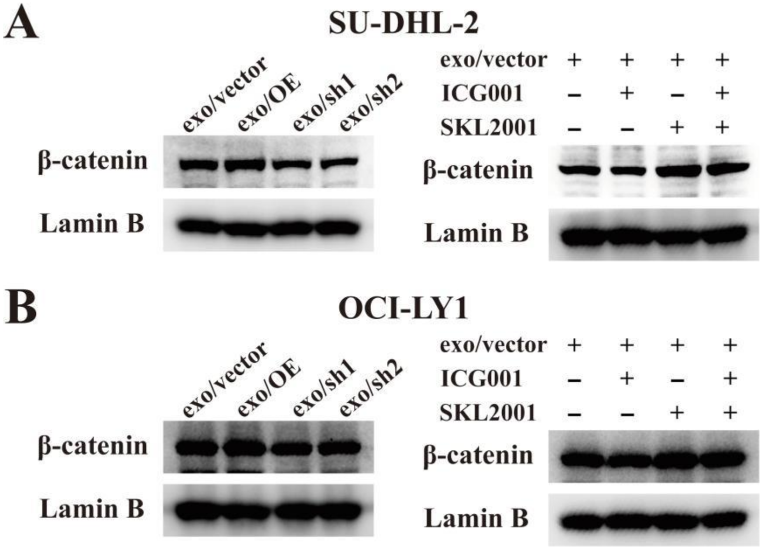

Supplement: Supplementary file 1 — Supplementary figures and tables. [file ijbsv20p0848s1.pdf]
